# Effectiveness of Contrast-Enhanced CT Venography 3D Reconstruction for Emergency Surgery in Non-Occlusive Mesenteric Ischemia: A Case Report

**DOI:** 10.70352/scrj.cr.25-0747

**Published:** 2026-03-25

**Authors:** Yasuo Suehiro, Hiromichi Fujii, Shoichi Ehara, Tomonori Yamamoto, Yosuke Takahashi, Kiyoshi Maeda

**Affiliations:** 1 Department of Intensive Care Medicine, Osaka Metropolitan University, Osaka, Osaka, Japan; 2 Department of Cardiovascular Surgery, Osaka Metropolitan University, Osaka, Osaka, Japan; 3 Department of Gastroenterological Surgery, Osaka Metropolitan University, Osaka, Osaka, Japan

**Keywords:** nonocclusive mesenteric ischemia, cardiac surgery, CT venography, surgical treatment, hemodialysis

## Abstract

**INTRODUCTION:**

Nonocclusive mesenteric ischemia (NOMI) is a lethal postoperative complication associated with a dismal prognosis. It generally causes hemodynamic instability, and thus prompt and appropriate management is required for both examination and treatment. We report a case of NOMI that was diagnosed by CT and angiography after cardiac surgery and was successfully treated by enterectomy.

**CASE PRESENTATION:**

A 59-year-old woman who had been on hemodialysis for 14 years underwent aortic and mitral valve replacement. On POD 16, the patient presented with severe abdominal pain associated with peritoneal signs. Based on the findings of no evident mesenteric occlusion, decreased enhancement in the intestine, and air in the intestinal wall with hepatic portal venous gas on contrast-enhanced CT images, NOMI was strongly suspected. The findings of scattered vascular stenosis in the superior mesenteric artery and poor enhancement of the intestinal parenchyma on subsequent angiography led to a definitive diagnosis of NOMI. Furthermore, the absence of enhancement of the superior mesenteric vein and intestinal parenchyma on the 3D-reconstructed images of the venous phase on enhanced CT, which were obtained after the angiography, led to the diagnosis of intestinal transmural ischemia and a decision for emergency exploratory laparotomy. During the surgery, discontinuous ischemia was observed in the small intestine from approximately 220 cm distal to Treitz’s ligament to the proximal side of the transverse colon. Therefore, a right hemicolectomy, partial ileal resection, and ileostomy were performed. New mesenteric ischemia was not observed postoperatively, and the patient was discharged on POD 53 after the laparotomy.

**CONCLUSIONS:**

NOMI is a rare but extremely severe disease entity, and its diagnosis is often delayed because it can be asymptomatic. NOMI often causes hemodynamic deterioration, and thus an appropriate therapeutic strategy should be promptly selected. In cases of suspected NOMI, angiography and contrast-enhanced CT are helpful for reaching a definitive diagnosis. In particular, 3D reconstructed images of the venous phase on enhanced CT are very useful for diagnosing intestinal transmural ischemia and reaching a decision for surgical intervention, which is considered feasible unless an intra-arterial vasodilator appears to exhibit its efficacy.

## Abbreviations

CHDF
: continuous hemodiafiltration
CRP
: C-reactive protein
HD
: hemodialysis
NOMI
: nonocclusive mesenteric ischemia
SMA
: superior mesenteric artery
SMV
: superior mesenteric vein
WBC
: white blood cell

## INTRODUCTION

Nonocclusive mesenteric ischemia (NOMI) is a disease characterized by acute intestinal ischemia without obvious mesenteric artery occlusion and has an extremely high mortality rate. Hemodialysis (HD) and the immediate phase after cardiac surgery are important potential risk factors for NOMI. Thus, prompt imaging examinations, such as CT and angiography, are mandatory when NOMI is suspected. In NOMI cases, spasmic changes in the mesenteric arteries are detected on both CT and angiography, but these findings do not assist in decision-making whether to conduct a laparotomy. Furthermore, a laparotomy is not indicated once the hemodynamic state deteriorates. Herein, we present a successful surgical case of NOMI in which the absence of enhancement of the superior mesenteric vein (SMV) on 3D-reconstructed CT images of the venous phase became a decisive finding for performing a laparotomy.

## CASE PRESENTATION

**Fig. 1:**
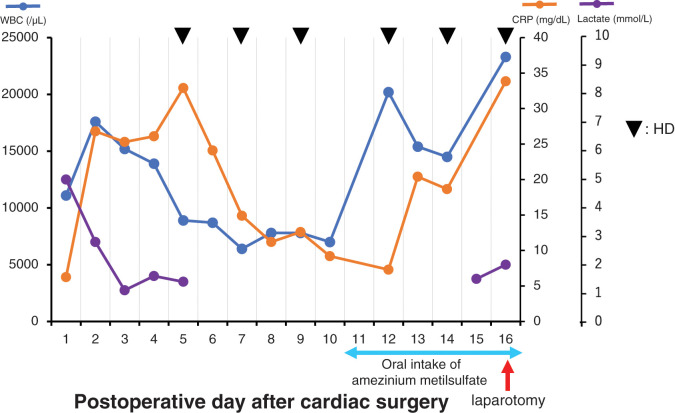
Changes in laboratory data between the cardiac surgery and the laparotomy. CRP, C-reactive protein; HD, hemodialysis; WBC, white blood cell

A 59-year-old woman who presented with dyspnea on exertion visited our hospital. The patient had both hypertension and type 2 diabetes mellitus, and had been receiving HD for 14 years due to diabetic nephropathy. On admission, transthoracic echocardiography showed normal contraction, severe mitral regurgitation, and aortic stenosis. Preoperative laboratory tests revealed no abnormal findings other than highly elevated values for blood urea nitrogen (54 mg/dL) and creatinine (3.4 mg/dL) because of chronic kidney disease. Thus, she successfully underwent mitral valve and aortic valve replacement. The cardiopulmonary bypass time and aortic clamp time were 342 and 242 min, respectively. The patient was started on continuous hemodiafiltration (CHDF) on POD 1 and was extubated on POD 3. The patient was weaned from CHDF on POD 5, discharged from the ICU on POD 6, and received regular HD thereafter. Administration of catecholamine was discontinued on POD 8. On POD 11, oral intake of amezinium metilsulfate was started because the patient showed a tendency toward low systolic blood pressure of 60–70 mmHg during HD sessions. Remarkably elevated serum white blood cell (WBC) count (20200/μL) and C-reactive protein (CRP) concentration (20.4 mg/dL) were observed on PODs 12 and 13, respectively (**Fig. 1**). However, further examinations were not undertaken because the clinical manifestations and other laboratory findings were unremarkable.

**Fig. 2:**
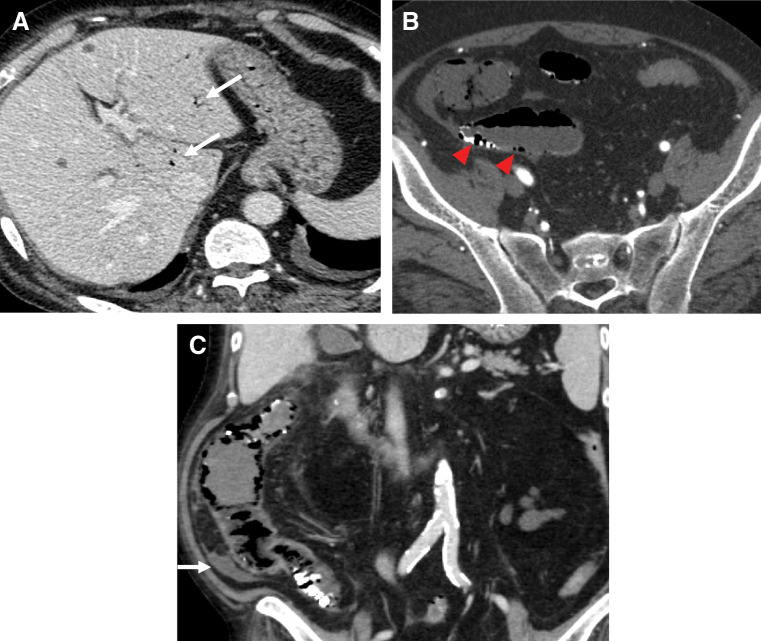
Contrast-enhanced CT findings. (**A**) Axial image showing the presence of hepatic portal venous gas (white arrows). (**B**) Axial image showing thinned intestinal wall and pneumatosis intestinalis of the small intestine (red arrowheads). **(C)** Coronal image showing thinned and poorly contrasted intestinal wall and bloody ascites (white arrow).

**Fig. 3:**
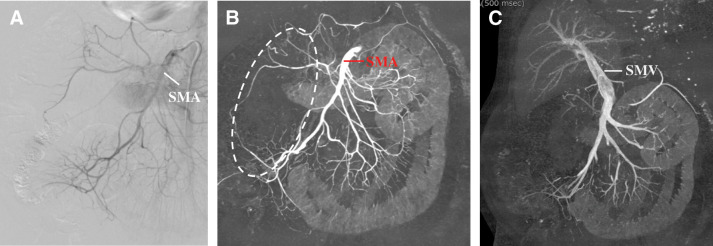
(**A**) Angiography findings. The SMA showed spasmic changes, and the middle colic artery, right colic artery, and ileocolic artery were poorly enhanced. (**B**, **C**) 3D-reconstructed images from the contrast-enhanced CT, which were obtained after the angiography. (**B**) Arterial phase. The intestinal parenchyma supplied by the middle colic artery, right colic artery, and ileocolic artery was poorly enhanced (area surrounded by the dotted white line). (**C**) Venous phase. The right colic vein was not enhanced. SMA, superior mesenteric artery; SMV, superior mesenteric vein

On POD 16, the patient complained of severe abdominal pain with peritoneal signs during HD. Laboratory tests revealed elevation of the serum WBC count (23300/μL), CRP concentration (33.85 mg/dL), and lactate concentration (2.0 mmol/L), with a reduced creatinine kinase concentration (12 U/L). Contrast-enhanced CT showed a lack of evident mesenteric occlusion, decreased enhancement in the intestine, hepatic portal venous gas, pneumatosis intestinalis of the small intestine, and bloody ascites (**Fig. 2**). Subsequent angiography demonstrated thickening and irregularities of the superior mesenteric artery (SMA), and poor enhancement of the intestinal parenchyma supplied by the middle colic artery, right colic artery, and ileocolic artery, leading to the diagnosis of NOMI (**Fig. 3**). Selective infusion of papaverine hydrochloride into the SMA was performed, but dilation of the SMA was not clearly observed. Finally, the absence of enhancement of the SMV and intestinal parenchyma was clearly confirmed on 3D-reconstructed images of the venous phase on enhanced CT, which were obtained after the angiography, and an emergency exploratory laparotomy was scheduled according to these findings.

**Fig. 4:**
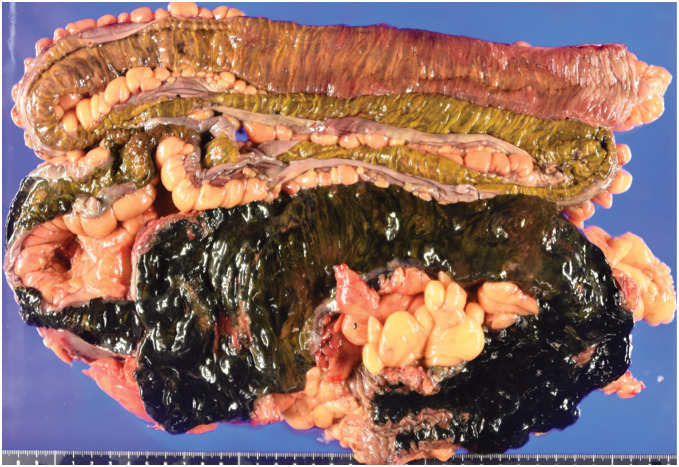
Resected intestine. The resected intestine was approximately 180 cm in length and showed discontinuous dark red color changes on the serosal side, indicating ischemia.

During surgery, dark red color changes were observed on the serosal side, indicating ischemia, together with skip lesions in the intestine from 220 cm distal to Treitz’s ligament to the proximal side of the transverse colon via the terminal ileum. A right hemicolectomy, partial ileal resection, and ileostomy were successfully performed, and selective infusion of papaverine hydrochloride into the SMA was continued postoperatively to ensure blood flow. The resected part of the intestine was approximately 180 cm in length (**Fig. 4**). The pathological findings, including inflammatory cell infiltration of the serosal surfaces, severe edema, hemorrhage, and congestion of the submucosa, were consistent with the features of NOMI, although the finding of thrombi in small veins within the mesentery was atypical for NOMI and may have arisen through disease progression.

The patient was safely extubated on POD 1 after the laparotomy. On POD 18 after the laparotomy, drainage treatment was performed for an infection of the abdominal wound, and on POD 24 after the laparotomy, an artificial cardiac pacemaker was implanted for a complete atrioventricular block caused by the cardiac surgery. The patient was discharged on POD 53 after the laparotomy.

## DISCUSSION

NOMI is a rare but life-threatening condition characterized by acute intestinal ischemia arising from spasmic changes in the mesenteric vessels without evident mesenteric artery occlusion, especially when the diagnosis and treatment are delayed. The potential risk factors for NOMI are older age, renal disease, HD, diabetes mellitus, catecholamine or vasopressor use, diuretics and digitalis use, hypotension, and cardiac disease or cardiac or abdominal surgery.^1)^ NOMI accounts for 20%–30% of all cases of acute mesenteric ischemia,^2)^ and its incidence rate after cardiovascular surgery has been reported to be <0.5%.^3)^ HD is a poor prognostic factor for NOMI because the intestinal mucosa is generally damaged by hypotension, hypovolemia, and vascular arteriosclerosis associated with HD. Recent studies have indicated that the frequency of NOMI is increasing in the HD population, with a frequency of 1.9% per patient-year,^4,5)^ and that HD-dependent patients have a 100-fold higher incidence of NOMI than non-HD-dependent patients.^6)^ The risk factors for NOMI in the perioperative period of cardiac surgery include advanced age (>70 years), onset of atrial fibrillation, use of diuretics, renal impairment, prolonged duration of procedure (>240 min), cardiopulmonary bypass time (>100 min), aortic clamp time (>60 min), postoperative intra-aortic balloon pumping, and elevated lactate concentration (>5 mmol/L).^7)^ Given these risk factors, the present case was considered to be at high risk for NOMI.

The clinical symptoms of NOMI are nonspecific; these include abdominal pain and distention due to loss of bowel movement. Some cases of NOMI remain asymptomatic, often leading to a delay in diagnosis. Laboratory findings commonly show the elevations of serum creatinine kinase, lactate dehydrogenase, WBC count, CRP, fibrin degradation products, and D-dimer values, although they are nonspecific markers.^8)^ In the present case, sudden elevations of the serum WBC count and CRP concentration were noted on days 1 and 2 after starting oral intake of amezinium metilsulfate, respectively, and the patient complained of abdominal pain on day 5 after initiating the intake. Vasopressor administration is another risk factor for NOMI, and these oral medications are considered highly associated with the onset of NOMI. In our patient, intestinal ischemia would already have been present when the WBC count and CRP concentration became elevated because NOMI sometimes lacks abdominal symptoms.

Spasmic changes in the mesenteric vessels, which arise at the expense of maintaining perfusion of the vital organs in a state of shock, lead to significantly reduced perfusion of the intestine and, consequently, in the worst situation, to transmural ischemia. Intestinal villi are susceptible to ischemia, and the small bowel is thought to be the splanchnic territory most inclined toward ischemia. This process triggers a systemic inflammatory response that contributes to the extremely high mortality rate of NOMI at >90%,^1)^ which is considered partly attributable to the delay in diagnosis.^8)^

Imaging examinations, including contrast-enhanced CT and angiography, are important for the diagnosis of NOMI. Both contrast-enhanced CT and angiography generally reveal the presence of occlusion in the mesenteric trunk, while contrast-enhanced CT additionally indicates the presence of thinned and poorly contrasted intestinal wall, smaller SMV, and ascites, and angiography additionally shows thickening and irregularities of the mesenteric arteries and a contrast effect in the intestinal parenchyma.^8,9)^ Regarding contrast-enhanced CT, the presence of the SMV and flow in its branches can be assessed visually using 3D reconstructed images of the venous phase. In the present case, during the arterial phase on the enhanced CT, vasospasm and reduced flow in the SMA and its branches were found, while disappearance or interruption of flow was not, which matches the conventional definition of NOMI. Thus, assessment of the degree of intestinal ischemia is considered difficult using only the mesenteric arterial distribution finding obtained from the arterial phase on the enhanced CT. Severe intestinal ischemia can be diagnosed based on a contrast defect in the intestinal parenchyma; however, this finding cannot be used to establish a diagnosis of transmural ischemia. Generally, venous flow from an ischemic area will not only decrease but disappear entirely over time, especially with transmural ischemia because of failure of capillary perfusion and venous outflow. Disappearance of venous flow strongly suggests the diagnosis of transmural, or irreversible, ischemia. Pneumatosis intestinalis, hepatic portal venous gas, and ascites on plain CT are crucial findings for ischemia; however, pneumatosis does not always indicate intestinal transmural ischemia,^10)^ and ascites can be seen in many abdominal disorders. Furthermore, if the presence of transmural ischemia is determined in that manner, angiography can be omitted. NOMI has a high mortality rate and requires an emergency treatment. While angiography is helpful, it is invasive and requires some time to complete. Furthermore, a long time may elapse before the examination can begin, possibly allowing hemodynamic deterioration. Therefore, the omission of angiography by establishing a diagnosis of transmural ischemia based on 3D-reconstructed CT images is considered advantageous for the patient.

The therapeutic options for NOMI generally include laparotomy and intra-arterial administration of a vasodilator. John et al.^4)^ demonstrated that these 2 therapeutic options vary depending on the duration of the ischemia: if the ischemia time is <6 h, intra-arterial administration of a vasodilator, such as papaverine or prostaglandin, may be feasible, but if the ischemia time is >12 h, a laparotomy may be needed for exploration and resection of the necrotic intestine. However, this strategy may not be suitable because the duration of NOMI at the onset of symptoms cannot be estimated exactly, as mentioned above. Although the effectiveness of transarterial vasodilator infusion has been confirmed,^1,11)^ it is only considered effective when initiated before severe ischemic changes occur. In terms of clinical manifestation, a laparotomy is highly recommended for the evaluation of the viability of the intestinal villi once peritoneal signs have appeared.^7,12)^ Some fatal cases of NOMI that underwent enterectomy after ineffective vasodilator infusion therapy have been reported.^13,14)^ In the present case, the absence of enhancement of the SMV on 3D-reconstructed CT images finally became a definitive finding for performing a laparotomy, although a laparotomy could have been considered mandatory based on the clinical manifestations only. Furthermore, the angiography could have been omitted if the 3D-reconstructed CT images of the venous phase had been obtained before the angiography was conducted. Importantly, the disappearance of venous flow would also be helpful to convince surgeons of the need for emergency laparotomy, and the clarity of 3D-reconstructed CT images will make this decision easier.

In HD-dependent patients, NOMI following cardiac surgery is considered a possibly fatal complication, although reports are limited and survival cases are rare. Furthermore, contrast-enhanced CT may be useful for predicting patient prognosis and assessing bowel viability in early-stage NOMI. However, further investigation with large numbers of cases is needed. For delayed diagnosis of suspected NOMI, we believe that prompt and appropriate examinations and interventions can lead to a favorable outcome, and that contrast-enhanced CT venography 3D reconstruction is useful for the diagnosis of intestinal transmural ischemia, which certainly requires an emergency laparotomy.

This study has a limitation. In the present case, the disappearance of the right colic vein branching from the SMV was visualized in 3D-reconstructed images of the venous phase; however, this is a qualitative evaluation. For quantitative evaluation, comparison of quantitative data, such as Hounsfield unit measurements between the SMV and PV or other vessels, is necessary, and the development of a semi-quantitative grading system for venous enhancement is desirable. Notably, retrospective measurement of Hounsfield units in the images in the present case was impossible. We hope that more cases of NOMI will be evaluated quantitatively in the future.

## CONCLUSIONS

NOMI is a rare but extremely severe postoperative complication of cardiac surgery, especially when diagnosis and treatment are delayed. As an early diagnosis is difficult to establish because NOMI is sometimes asymptomatic, clinicians should therefore pay attention to changes in laboratory values and thoroughly assess the possibility of critical complications, including NOMI. Prompt and appropriate management is required because NOMI generally causes hemodynamic instability. Transmural ischemia is a decisive finding for conducting a laparotomy, and 3D-reconstructed images of the venous phase on enhanced CT can inform the presence of SMV flow, loss of which indicates transmural ischemia. If transmural ischemia is distinctly detected in the venous phase on enhanced CT, angiography may be omitted, thereby allowing immediate performance of a laparotomy before the hemodynamic state deteriorates. We believe that early diagnosis and prompt management are crucial for improving patient outcomes, and that a laparotomy is warranted when ischemia is suspected to be transmural.

## DECLARATIONS

### Availability of data and materials

The data will be shared on a reasonable request to the corresponding author.
